# Association between Axial Length to Corneal Curvature Radius Ratio and Myopia in Adult Patients

**DOI:** 10.1155/2024/4981095

**Published:** 2024-02-28

**Authors:** Yanyun Fan, Yikeng Huang, Xionggao Huang

**Affiliations:** Department of Ophthalmology, The First Affiliated Hospital of Hainan Medical University, Haikou 570102, Hainan Province, China

## Abstract

**Purpose:**

To analyze the distribution characteristics of axial length to corneal curvature radius ratio (AL/CR) and other ocular biometric parameters in adult myopia patients and their association with myopia.

**Methods:**

A cross-sectional study was conducted among patients with no eye diseases except ametropia who attended the optometry clinic of the First Affiliated Hospital of Hainan Medical College from January 2022 to June 2022. In total, 187 eyes (right eye) of 187 myopic patients aged 18–35 years were selected by random sampling. Based on the results of spherical equivalent (SE, (D)) obtained by postdilation optometry, all subjects were divided into three groups: mild myopia (≤−0.50D and >−3.00D, 42 eyes), moderate myopia (≤−3.00D and >−6.00D, 80 eyes), and high myopia (≤−6.00D, 65 eyes). The axial length (AL), corneal curvature radius (CR), and AL/CR were measured and compared between the three groups. The association between AL and AL/CR of the eye and SE was analyzed by multiple linear regression. Also, the predictive ability of AL/CR for high myopia was investigated by ROC curve.

**Results:**

There were no statistically significant differences in age, gender, or intraocular pressure between the three groups. The mean values of AL/CR in mild, moderate, and high myopia groups were 3.17 ± 0.06, 3.31 ± 0.08, and 3.43 ± 0.10, respectively, and the difference between the groups was statistically significant (*P* < 0.001). Linear regression analysis showed that both AL and AL/CR were strongly negatively correlated with SE (*P* < 0.05), while CR had a weak positive correlation with SE without statistically significant differences (*P* > 0.05). The adjusted linear regression equation shows that for every 0.1 unit increase in AL/CR, SE increases by 1.54 D. Compared with 0.830 (95% confidence interval: 0.769 to 0.900) for AL, the area under ROC curve of AL/CR was 0.896 (95% confidence interval: 0.851 to 0.941), indicating that the diagnostic value of AL/CR for high myopia was higher than that of AL (*P* <  0.01). When the Youden index reached its maximum (0.626), the AL/CR cutoff point was 3.309, and the sensitivity and specificity were 0.954 and 0.672, respectively.

**Conclusion:**

This study showed that AL and AL/CR in adult myopia patients were significantly negatively correlated with SE, and the corralation between AL/CR and SE is greater than that between AL and SE. Therefore, AL/CR can be used to analyze the dynamic changes of SE in the development of adult myopia independently of optometry on a certain basis, and it is especially suitable for the diagnosis of high myopia in adults. This trial is registered with ChiCTR2300069070.

## 1. Introduction

In recent years, the incidence of myopia has been high all over the world, especially among young people in East Asia for whom the prevalence of myopia can reach 80%∼90% [1]. The etiology of myopia is complex and the current research consensus is that both genetic and environmental factors contribute to the development of myopia [2]. For each individual, although genetic factors usually remain unchanged [3], their living environment is different and can change throughout life [4]. Therefore, the status and severity of myopia are likely to change accordingly, and the biological parameters of the eye may change in the development of myopia [5].

The current gold standard for the diagnosis of myopia and its severity is mydriatic refraction (cycloplegic refraction) [6], but in some cases, such as angle-closure glaucoma, patients are unable to undergo this examination and alternative means are needed [7]. Studies have confirmed that axial length (AL) is the most important ocular biological parameter affecting refractive status, while the association between corneal curvature radius (CR) and refractive state is inconsistent [8, 9]. Grosvenor found for the first time that there is a positive correlation between AL and CR, that is, when AL becomes longer, CR also becomes larger [10]. When AL gradually increases and leads to the focus of parallel light before the retina, the eye tends to develop myopia [11]. At this time, the cornea plays a regulatory role, making the eye return to emmetropia as much as possible through its compensation effect of flattening (CR increases). However, myopia occurs when the axial growth of the eye exceeds a certain limit and the corneal flattening effect cannot compensate for the myopia changes [12]. Therefore, axial length to corneal curvature radius ratio (AL/CR) can reflect the refractive state of myopia to a certain extent. Many previous studies, based on children and adolescents aged 3–18 years, have found that the limit of corneal compensation is reached when AL/CR ≥ 3, and AL/CR is associated with SE [13, 14]. However, as far as we know, there are few studies on AL/CR in adult myopia patients.

The purpose of this study was to investigate the distribution characteristics of the biological parameters of the eyes, such as AL, CR, and AL/CR, and their association with myopia in adult patients. This study is expected to provide a new idea for clinical evaluation of myopia status and severity in adult patients.

## 2. Materials and Methods

### 2.1. Study Design and Subjects

This study was a single-center cross-sectional study. We collected information of adult patients with myopia who visited the optometry clinic of the First Affiliated Hospital of Hainan Medical College from January to June 2022 for analysis. Subjects with a previous history of keratopathy or ocular surgery were excluded. Finally, a total of 187 patients with myopia (187 eyes) aged from 18 to 35 years old were included in this study. Meanwhile, those wearing hard contact lenses were required to stop wearing them for more than 4 weeks, and those wearing soft contact lenses were required to stop wearing them for more than 2 weeks. Based on SE of the right eye, subjects were divided into three groups: mild myopia (group A, ≤−0.50D and >−3.00D, 42 eyes), moderate myopia (group B, ≤−3.00D and >−6.00D, 80 eyes), and high myopia (group C, ≤−6.00D, 65 eyes). This study is in accordance with the relevant requirements of the Declaration of Helsinki of the World Medical Association.

### 2.2. Measurement of Ocular Biological Parameters

All patients received ophthalmic examinations from 8:00 a.m. to 12:00 a.m. First, the slit lamp examination, naked eye visual acuity, intraocular pressure (IOP), AL, CR, central corneal thickness (CCT), lens thickness (LT), anterior chamber depth (ACD), and other ocular biological parameters were performed under natural pupil. After that, mydriatic refraction was performed to determine the SE of myopia patients (the result was expressed as SE = (spherical value + column value)/2, unit D). The LS900 optical biometrics instrument (Crystal Star 900) was used to measure AL while the Pentacam corneal topographic instrument was used to measure CR (Rf and Rs on the anterior surface of the cornea were obtained, and the average corneal curvature radius Rm = (Rf + Rs)/2), CCT, LT, and ACD. Finally, AL/CR was calculated. All of the above examinations were performed by the same ophthalmologist. A total of 3 measurements were taken for each patient, and the average values were taken as the final results. Since CR and the corneal curvature *K* value can be converted from each other through the formula (CR = 1000 *∗* (*n*2 − *n*1)/*K* (air refraction index *n*1 = 1.0, corneal curvature refractive index *n*2 = 1.3375)) and it is believed that both CR and *K* value represent corneal radians, only CR is used in this article.

### 2.3. Statistical Analysis

SPSS statistical software 26.0 (Chicago, IL, USA) was used for data analysis in this cross-sectional study. Normality tests were performed using the Shapiro–Wilk test for quantitative variables. If the data conformed to the normal distribution, mean ± standard deviation was used for data presentation and one-way ANOVA was applied to the comparison among the three groups with Bonferroni test being used for pairwise comparison afterwards. If the data did not conform to the normal distribution, median (P25, P75) was used for data presentation and Kruskal–Wallis *H* test was applied to the comparison between groups with Bonferroni test being used for pairwise comparison afterwards. For qualitative data, absolute number (percentage) was used for data presentation and chi-square test was used for comparison between groups. Spearman correlation coefficient was used to analyze the correlation between ocular biological parameters and SE. Linear regression was used to fit the association between biological parameters of the eye and SE. Age, sex, and intraocular pressure were adjusted in the multivariate model. Continuous AL/CR was transformed into a quartile form to construct a linear regression model as a sensitivity analysis. Receiver operating characteristic curve (ROC curve) was used to analyze the predictive effect of AL and AL/CR on high myopia in myopic patients. The area under the ROC curve and its 95% confidence intervals were also calculated. PASS software was used (version 15.0) to calculate the posterior statistical power. A power greater than 0.8 was considered adequate to detect a positive result.

## 3. Results

### 3.1. Ocular Biological Parameters of Myopia Patients

The median age of 187 subjects was 26 (17, 43) years old, including 99 males (52.94%) and 88 females (47.06%). The median IOP was 14 (12, 16) mmHg. There were no significant differences in age (*P*=0.262), gender (*P*=0.301), and intraocular pressure (*P*=0.403) among the three groups. For ocular biological parameters, AL, AL/CR, and SE showed statistically significant differences among the three groups (*P* < 0.001), while CR, CCT, LT, and ACD did not (*P* > 0.05). A two-by-two comparison was performed by ANOVA among the three groups, and only AL, AL/CR, and SE were significantly different among the three groups (*P* < 0.05) (Table 1). Under the hypothesis test level *α* = 0.05 and the minimum within-group sample size *n* = 42, the statistical power (1-*β*) of the ANOVA for AL and AL/CR was greater than 0.999, indicating that there was sufficient power to find positive results.

### 3.2. Association of Ocular Biological Parameters and SE

**Figure 1:**
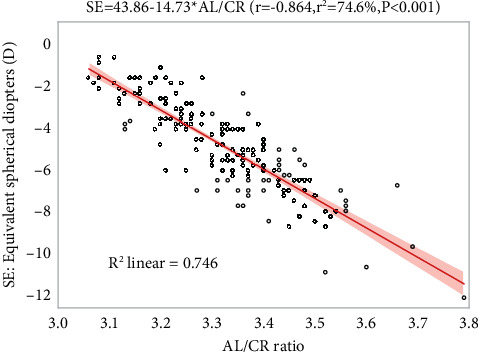
Univariate linear regression model and 95% confidence interval for AL/CR and SE. SE = spherical equivalent (D); AL/CR = axial length to corneal curvature radius ratio.

There was a negative linear correlation between AL and SE (*r* = −0.727, *r*^2^ = 52.8%, *P* < 0.001). According to the linear regression model, when AL increases by 1 mm, SE decreases by −1.497D, that is, myopia increases by 1.497D. Similarly, there was a negative linear correlation between AL/CR and SE (*r* = −0.864, *r*^2^ = 74.6%, *P* < 0.001). According to the linear regression model, for every 1 unit increase in AL/CR, SE decreased by −15.412D. However, there was no evidence of linear correlation between CR and SE (*r* = 0.021, *r*^2^ = 0.05%, *P*=0.771), Table 2 and Figure 1.

### 3.3. Sensitivity Analysis for the Association of AL/CR and SE

In order to further clarify the association between AL/CR and SE, continuous AL/CR was transformed into a quartile form to construct a linear regression model. Compared with Quantile1 (<3.23), the regression coefficients of Quantile2 (3.23–3.32), Quantile3 (3.32–3.41), and Quantile4 (>−3.41) were −1.759, −3.137, and −4.934, respectively, with statistically significant differences (*P* < 0.001), Table 3.

### 3.4. ROC Curve Analysis: The Predictive Ability of AL and AL/CR for High Myopia

**Figure 2:**
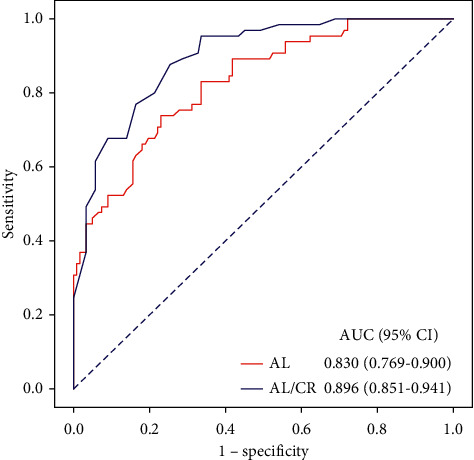
ROC curve of AL and AL/CR in the diagnosis of high myopia. AL = axial length (mm); AL/CR = axial length to corneal curvature radius ratio.

The predictive ability of AL/CR for high myopia was investigated by ROC curve. The area under ROC curve of AL and AL/CR was 0.830 (95% CI: 0.769–0.900) and 0.896 (95% CI: 0.851–0.941), respectively, indicating that AL/CR had a higher diagnostic value than AL (*P* < 0.01). In the ROC curve of AL/CR, the cutoff point of the maximum Youden index (0.626) was 3.309, the sensitivity was 0.954, and the specificity was 0.672, Table 4 and Figure 2. These results indicate that AL/CR > 3.309 has a high diagnostic value for high myopia and can be used as a monitoring point.

## 4. Discussion

In this study, the patients were divided into three groups: mild myopia (≤−0.50D and >−3.00D), moderate myopia (≤−3.00D and >−6.00D), and high myopia (≤−6.00D), according to SE. Only AL (*P* < 0.001) and AL/CR (*P* < 0.001) showed statistically significant differences among the three groups, and the values of AL and AL/CR increased with the increase of myopia severity. However, there was no significant difference in CR, CCT, LT, and ACD among the three groups. The results of this study are slightly different from those of Chu et al. [15]. The study pointed out that not only AL/CR and AL but also CR had statistically significant differences between the high myopia group and the non-high myopia group (*P* < 0.05) [15]. This may be due to differences in the average age of the sample and the grouping strategy. Unlike the majority of participants in our study who were myopic adults (ages 17–43), Chu et al. included young myopic patients (ages 7–48) in their study. Other studies of young people with myopia have reached similar conclusions. For example, a study of 308 myopic children (ages 7 to 16) found significant differences in AL, AL/CR, and CR among groups with different degrees of myopia [12, 15]. This suggests that age may have a significant effect on the distribution of ocular biological parameters. In fact, studies have confirmed that AL and CR in children constantly change with the gradual completion of eyeball development. However, in adults, after the development of the eyeball is basically complete, the increase of AL can continue until the age of 30, but other refractive components usually do not change, which is considered to be an important cause of axial myopia. Since the association between AL or CR and myopia seems to be unstable, this may have contributed to the insignificant difference in CR in our study. In addition, recent studies have shown that AL/CR in adolescents and children is more closely related to myopia than AL alone, so attention should be paid to exploring AL/CR in adults, as it may be a more stable indicator for evaluating the progression of myopia. Our study further verifies the possibility that AL/CR also indicates the severity of myopia in adults with myopia.

Spearman correlation coefficient was used to explore the correlation between AL and AL/CR and SE in this study. The results showed that although both AL and AL/CR were negatively correlated with SE, the correlation between the latter (*r* = −0.864, *r*^2^ = 74.6%, *P* < 0.001) and SE was stronger than the former (*r* = −0.727, *r*^2^ = 52.8%, *P* <  0.001). Then, the correlation between AL, CR, AL/CR, and SE was further analyzed by linear regression. After adjusting for confounding factors such as age, sex, and IOP, it was found that only AL and AL/CR independently affected SE. Specifically, for every 1 unit increase in AL/CR, SE decreased by 15.41D, that is, myopia increased by 15.41D, showing a strong negative correlation between them. In contrast, SE only decreased by 1.50D for every 1 unit increase in AL. In other words, a small change in AL/CR corresponds to a significant change in refractive status, so AL/CR can be used as a highly sensitive indicator to indicate or predict the progression of myopia. In a study of adults with myopia in Nigeria, it was concluded that for every 1 unit change in AL/CR, there was an 8.89D change in myopia (*r*^2^ = 51%, *P* <  0.001), while for every 1 mm change in AL, myopia changed by 0.77D (*r*^2^ = 39%, *P* <  0.001) [16]. Although the results of this study are the same as ours in trend, there are still differences in values. The differences could be due to racial differences or differences in the age of the sample. This study was based on 350 samples, of which only 83 (54.97%) were 30 years old or younger, while 179 (95.72%) of the 187 samples in our study were younger than 30 years old. Previous studies have shown that AL plays a major role in the development of myopia within the age of 30. This may be one of the reasons why the results of this study are numerically smaller than ours.

In order to further evaluate the diagnostic value of AL/CR on high myopia, ROC curve analysis was adopted. The results showed that the sensitivity and specificity of AL/CR for predicting high myopia were 0.954 and 0.672, respectively, while the sensitivity and specificity of AL were 0.738 and 0.770. This indicates that AL/CR has a lower rate of misdiagnosis and missed diagnosis than AL in predicting high myopia, that is, it has a better predictive ability. It is obvious that high myopia is associated with an increased risk of potentially sight-threatening ocular conditions, such as myopic macular degeneration, glaucoma, cataract, and retinal detachment. Also, AL/CR > 3.309 can be a warning point for high myopia in order to intervene early and slow down the progression of myopia and ultimately reduce the ocular disease associated with high myopia, so AL/CR may help to objectively measure the risk of high myopia.

AL/CR has shown an advantage in the diagnosis of high myopia for several reasons. Firstly, AL/CR provides a reliable alternative to the diagnosis of high myopia in patients who are unable to undergo cycloplegic refraction. In order to ensure the accuracy of SE measurement, optometry is usually performed after cycloplegia [17]. However, cycloplegic refraction is not suitable for all patients, such as patients with acute angle-closure glaucoma who may have a further increase in intraocular pressure after cycloplegia [17]. In this case, AL/CR measurement can be used as a reliable alternative method to assess the presence of high myopia in these patients. Secondly, AL/CR can partially reveal the mechanical properties of the eyeball and provide a more comprehensive reflection of the shape of the eyeball. As myopia progresses, both the cornea and sclera tend to expand, causing the axial length of the eyeball to increase and the cornea to flatten [18, 19]. This may be due in part to the thinning of collagen fibers in the posterior pole of the sclera leading to deterioration of biomechanical properties [20, 21]. However, the extent of expansion of the cornea and sclera can vary among individuals, resulting in different final shapes of the eyeball. When the eyeball is approximately spherical, the AL/CR value tends to be smaller, while the oval eyeball corresponds to a larger AL/CR value, and the latter is often closely related to retinal detachment, retinoschisis, and macular damage [22, 23]. In fact, in high myopia, the shape of the eyeball may change even when the axial length remains constant [21, 24–26]. Therefore, AL/CR can better reflect the pathological changes of ocular morphology caused by myopia progression than AL and can be used as a more reliable indicator to diagnose myopia and measure the risk of fundus lesions in patients with high myopia. Thirdly, AL/CR can also serve as an indicator of peripheral retinal defocus. Previous studies have demonstrated that peripheral defocus plays a role in regulating axial length growth [20]. Hyperopic defocus tends to elongate the axial length, whereas myopic defocus tends to inhibit its growth [27]. For individuals with a larger AL/CR, indicating a greater curvature of the posterior eyeball relative to the cornea, light entering the eye is more likely to create hyperopic defocus around the retina. We posit that this differential effect on retinal defocus is one of the reasons why AL/CR proves to be a more advantageous diagnostic measure for identifying individuals at risk of developing high myopia.

Our study innovatively confirmed the association between AL/CR and myopia in a young adult sample, which was consistent with previous research results based on children and adolescents, indicating a significant negative correlation between AL/CR and SE and also indicating that AL/CR is an independent factor affecting the development of myopia. More importantly, our results provide a promising method for refractive screening in myopic patients who are allergic to mydriatic drugs or whose eye structure does not allow mydriasis. In the future, it is expected to establish a relatively accurate, reliable, convenient, and widely applicable alternative refractive examination method based on AL/CR.

However, this study also has the following limitations. ① Given the cross-sectional nature of this study, it is necessary to acknowledge its limitation in establishing a causal relationship between AL/CR and high myopia. Future longitudinal studies are highly warranted in order to examine this issue in more depth. ② A small number of subjects were collected in this study, and most of them were from refractive surgery clinics. Patients with moderate and high myopia accounted for a high proportion in this study, while mild and moderate myopia were more common in the normal population, which may cause our results to be less general. ③ Data collection was incomplete and there were other confounding factors that could be ruled out, such as height, weight, body mass index, and oxygen saturation. ④ Finally, given the ethnic heterogeneity of myopia, the potential bias caused by the selective sampling of subjects from the same hospital and geographic location may limit the generalizability of the conclusions of this study to the broader population. Therefore, the conclusions of our study should be interpreted with caution, and it is necessary to conduct multicenter studies across regions in the future. Therefore, a multiethnic, multicenter study is necessary to verify our findings.

## 5. Conclusion

This study showed that AL and AL/CR were significantly negatively correlated with SE in adult myopia patients, and the correlation between AL/CR and SE is greater than that between AL and SE. AL/CR can be used as an alternative refractive status evaluation method for mydriatic refraction and has good specificity and sensitivity for the diagnosis of high myopia in adults. In the future, large sample size and multicenter studies are needed to further confirm this conclusion and guide the clinical application of AL/CR.

## Figures and Tables

**Table 1:** Comparison of general data and ocular biological parameters between the three groups.

Variables	Mild myopia (*n* = 42)	Moderate myopia (*n* = 80)	High myopia (*n* = 65)	F/H/*χ*^2^	*P*
Age, median (P_25_, P_75_) (year)	18 (18, 21.75)	21 (18, 24.25)	19 (18, 22)	2.68^c^	0.262
Men, *n* (%)	18 (42.86%)	46 (57.50%)	35 (53.85%)	2.40^b^	0.301
Intraocular pressure, median (P_25_, P_75_) (mmHg)	13 (11, 15.75)	13.50 (12, 16.25)	14 (12, 16)	1.82^c^	0.403
SE, median (P_25_, P_75_) (D)	−2.25 (−2.75, −1.75)	−4.38 (−5.5, −4)^*∗*^	−7.25 (−8, −6.5)^*∗*^^†^	161.85^c^	<0.001
AL, mean ± SD (mm)	24.81 ± 0.83	25.74 ± 0.83^*∗*^	26.78 ± 1.05^*∗*^^†^	60.87^a^	<0.001
CR, mean ± SD (mm)	7.83 ± 0.27	7.78 ± 0.23	7.80 ± 0.23	0.45^a^	0.637
AL/CR, mean ± SD	3.17 ± 0.06	3.31 ± 0.08^*∗*^	3.43 ± 0.10^*∗*^^†^	128.21^a^	<0.001
CCT, mean ± SD (*μ*m)	553.6 ± 27.46	543.11 ± 29.51	549.46 ± 31.47	1.89^a^	0.154
LT, mean ± SD (mm)	3.46 ± 0.27	3.51 ± 0.22	3.48 ± 0.18	0.86^a^	0.427
ACD, mean ± SD (mm)	3.23 ± 0.27	3.28 ± 0.25	3.25 ± 0.26	0.53^a^	0.590

^*∗*^ *P* < 0.05 compared to mild myopia; ^†^*P* < 0.05 compared to moderate myopia. ^a^*F* value of one-way ANOVA; ^b^chi-square value; ^c^*H* value of KW *H* test. SE = spherical equivalent (D), determined by mydriatic refraction; AL = axial length (mm); CR = corneal curvature radius (mm); AL/CR = axial length to corneal curvature radius ratio; CCT = central corneal thickness (*μ*m); LT = lens thickness (mm); ACD = anterior chamber depth (mm).

**Table 2:** Association of ocular biological parameters and SE.

Variables	Model 1				Model 2			
	*β*	S.E.	*t*	*P*	*β*	S.E.	*t*	*P*
AL (mm)	−1.361	0.095	−14.399	<0.001	−1.497	0.099	−15.154	<0.001
CR (mm)	0.197	0.675	0.292	0.771	0.341	0.704	0.485	0.629
AL/CR	−14.734	0.632	−23.315	<0.001	−15.412	0.644	−23.917	<0.001
CCT (*μ*m)	0.002	0.005	0.382	0.703	0.002	0.005	0.371	0.711
LT (mm)	−0.955	0.737	−1.297	0.196	−0.747	0.774	−0.964	0.336
ACD (mm)	−0.153	0.624	−0.245	0.807	0.13	0.671	0.194	0.847

Model 1, unadjusted; model 2, adjusted for age, gender, and intraocular pressure. *β* represents unstandardized beta; S.E. represents standard error. SE = spherical equivalent (D); AL = axial length (mm); CR = corneal curvature radius (mm); AL/CR = axial length to corneal curvature radius ratio; CCT = central corneal thickness (*μ*m); LT = lens thickness (mm); ACD = anterior chamber depth (mm).

**Table 3:** Sensitive analysis: linear regression of quartile form AL/CR and SE.

AL/CR	Model 1				Model 2			
	*β*	S.E.	*t*	*P*	*β*	S.E.	*t*	*P*
Quantile1 (<3.23)	Ref	—	—	—	Ref	—	—	—
Quantile2 (3.23–3.32)	−1.684	0.273	−6.178	<0.001	−1.759	0.273	−6.452	<0.001
Quantile3 (3.32–3.41)	−3.056	0.273	−11.212	<0.001	−3.137	0.276	−11.355	<0.001
Quantile4 (>−3.41)	−4.796	0.274	−17.498	<0.001	−4.934	0.281	−17.529	<0.001

Model 1, unadjusted; model 2, adjusted for age, gender, and intraocular pressure. *β* represents unstandardized beta; S.E. represents standard error. SE = spherical equivalent (D); AL/CR = axial length to corneal curvature radius ratio.

**Table 4:** Area under ROC curve of AL and AL/CR.

Variables	AUC	95% CI	Specificity	Sensitivity	Youden's index
AL (mm)	0.830	0.769–0.900	0.770	0.738	0.508
CR (mm)	0.502	0.415–0.589	0.484	0.569	0.053
AL/CR	0.896	0.851–0.941	0.672	0.954	0.626

AUC: the area under ROC curve; SE = spherical equivalent (D); AL = axial length (mm); AL/CR = axial length to corneal curvature radius ratio.

## Data Availability

The data used to support the findings of this study are available from the corresponding author upon reasonable request.
